# Predicting microRNA precursors with a generalized Gaussian components based density estimation algorithm

**DOI:** 10.1186/1471-2105-11-S1-S52

**Published:** 2010-01-18

**Authors:** Chih-Hung Hsieh, Darby Tien-Hao Chang, Cheng-Hao Hsueh, Chi-Yeh Wu, Yen-Jen Oyang

**Affiliations:** 1 Department of Computer Science and Information Engineering, National Taiwan University, Taipei 106, Taiwan; 2 Department of Electrical Engineering, National Cheng Kung University, Tainan, 70101, Taiwan; 3 Institute of Networking and Multimedia, National Taiwan University, Taipei 106, Taiwan; 4 Center for Systems Biology and Bioinformatics, National Taiwan University, Taipei 106, Taiwan

## Abstract

**Background:**

MicroRNAs (miRNAs) are short non-coding RNA molecules, which play an important role in post-transcriptional regulation of gene expression. There have been many efforts to discover miRNA precursors (pre-miRNAs) over the years. Recently, *ab initio *approaches have attracted more attention because they do not depend on homology information and provide broader applications than comparative approaches. Kernel based classifiers such as support vector machine (SVM) are extensively adopted in these *ab initio *approaches due to the prediction performance they achieved. On the other hand, logic based classifiers such as decision tree, of which the constructed model is interpretable, have attracted less attention.

**Results:**

This article reports the design of a predictor of pre-miRNAs with a novel kernel based classifier named the generalized Gaussian density estimator (G^2^DE) based classifier. The G^2^DE is a kernel based algorithm designed to provide interpretability by utilizing a few but representative kernels for constructing the classification model. The performance of the proposed predictor has been evaluated with 692 human pre-miRNAs and has been compared with two kernel based and two logic based classifiers. The experimental results show that the proposed predictor is capable of achieving prediction performance comparable to those delivered by the prevailing kernel based classification algorithms, while providing the user with an overall picture of the distribution of the data set.

**Conclusion:**

Software predictors that identify pre-miRNAs in genomic sequences have been exploited by biologists to facilitate molecular biology research in recent years. The G^2^DE employed in this study can deliver prediction accuracy comparable with the state-of-the-art kernel based machine learning algorithms. Furthermore, biologists can obtain valuable insights about the different characteristics of the sequences of pre-miRNAs with the models generated by the G^2^DE based predictor.

## Background

MicroRNAs (miRNAs) are short RNAs (~20-22 nt) that direct post-transcriptional regulation of target genes by arresting the translation of mRNAs or by inducing their cleavage [1]. Since the initial discovery of miRNAs in *Caenorhabditis elegans*, RNA molecules are regarded as not only a carrier of gene information, but also a mediator of gene expression [2,3]. Currently, 9539 experimentally verified miRNAs have been collected in the miRBase database [4].

Experimental miRNA identification is accomplished by directional cloning of endogenous small RNAs [5]. Considering both the time and cost of experimental methods, many computational approaches have been proposed [6]. The mature miRNAs is cleaved from a 70-120 nt precursor (pre-miRNA) with a stable hairpin structure. Identifying this specific structure has became an important step in analyzing miRNAs [1]. The earliest computational approaches for discovering pre-miRNAs are mainly based on comparative techniques and can only discover pre-miRNAs that are closely homologous to known miRNAs [7-10]. Alternatively, scientists have resorted to *ab initio *approaches to discover pre-miRNAs based on the characteristics of their secondary structures [11-15]. The *ab initio *approaches based predictors are more generally applicable than those that are based on homology searches, since the *ab initio *approaches do not rely on the existence of homologues. As a result, design of the *ab initio *approaches based predictors has attracted more attention in recent years.

The basis of the *ab initio *approaches is to design a coding scheme that maps the sequence and structure characteristics of pre-miRNAs into distinguishable patterns of feature vectors. Then, a supervised learning algorithm, also commonly referred to as data classification, is invoked to discover pre-miRNAs in the query RNA sequence based on the associated feature vectors. In recent years, the design of the coding scheme for characterizing pre-miRNAs has been extensively studied and several different schemes, including base pairing propensity [16], folding energy [17], base pair distance [18], and degree of compactness [19], have been proposed. On the other hand, most people working on this subject have employed the existing kernel based data classification algorithms such as the hidden Markov model (HMM) [20,21], the support vector machine (SVM) [22,23], and the kernel density estimator [15] to build the predictors due to the superior prediction performance delivered by these algorithms [24]. Nevertheless, conventional logic based data classification algorithms such as decision trees [25,26] and decision rules [27,28] continue to play a major role in some applications due to the *interpretability *of the logic rules identified by these algorithms. Such a summarized view of the characteristics and distribution of the data set further provides valuable insights about the relations among different features and is highly desirable for in-depth analysis of pre-miRNAs.

Aiming to provide the desirable functionalities of both the kernel based and the logic based data classification algorithms, the study presented in this article has exploited the generalized Gaussian density estimator (G^2^DE) that we have recently proposed [29]. The G^2^DE identifies a small number of generalized Gaussian components to model the distribution of the data set in the vector space. As a result, the user can examine the parameter values associated with these of Gaussian components to obtain an overview picture of the distribution of the data set. Furthermore, through in-depth analysis of the parameter values, the user can obtain valuable insights about the relations among different features.

## Results and discussion

This section first describes the overall scheme of using G^2^DE to analyze pre-miRNAs. Each step of the analysis procedure is further elaborated in the Methods section. Next, the prediction performance of the employed classification algorithm is evaluated and compared with four classification algorithms. A demonstrative analysis is also presented to investigate the interpretability of the employed classification algorithm.

### Using G^2^DE to analyze pre-miRNAs

This work uses only sequence information to identify pre-miRNAs from pseudo hairpins, which are RNA sequences with similar stem-loop features to pre-miRNAs but containing no mature miRNAs. In this method, each RNA sequence is represented as a feature vector. The characteristics used to generate the feature vector, including sequence composition, folding energy and stem-loop shape, have been shown to be useful for predicting pre-miRNAs in previous studies [17,18,30]. The main task carried out during the learning process of this method is to construct two mixture models of generalized Gaussian components for summarizing pre-miRNAs and pseudo hairpins in the vector space. We model this learning process as a large-parameter-optimization problem (LPOP) and employ an efficient optimization algorithm, Ranking-based Adaptive Mutation Evolutionary (RAME) [31], to decide the parameters associated with each generalized Gaussian component. Finally, the models learnt through the LPOP process are used to predict whether a query RNA sequence is a pre-miRNA. Furthermore, the constructed model of G^2^DE comprises a small number of generalized Gaussian components and is capable of detecting the sub-clusters or sub-classes of the data set. This study utilizes this feature of G^2^DE to develop a two-stage analysis where the first stage uses G^2^DE to partition the data set while the second stage uses G^2^DE to investigate each of the partitioned subsets.

### Prediction performance

The present approach is evaluated using two datasets, HU920 and HU424, combined with four feature sets. See the Methods section for details of the datasets and the feature sets. The prediction performance is compared to two kernel based classifiers, SVM and RVKDE, and two logic based classifiers, C4.5 and RIPPER. The parameters for each classifier are determined by maximizing the prediction accuracy of ten-fold cross-validation on the HU920. A prediction is performed by using the HU920 dataset to predict the HU464 dataset with the selected parameters.

The employed G^2^DE classifier is compared with two kernel based classifiers, SVM and the relaxed variable kernel density estimator (RVKDE) [32]. The SVM is a commonly used classifier because of its prevailing success in diverse bioinformatics problems [14,33,34], while the RVKDE has been shown to have advantages for predicting species-specific pre-miRNAs [15]. Two well-known logic based classifiers, C4.5 [35] and RIPPER [36], are also included as representatives of logic based classification algorithms.

As shown in Table 1, the prediction accuracies of one-stage G^2^DE are 80.39%, 92.03%, 91.60% and 78.66% with different feature sets. The two-stage G^2^DE further improve the prediction accuracies and achieves the best average accuracy of 86.58%. Though the number of kernels increases from one-stage to two-stage G^2^DE, it is still much less than the other kernel based classifiers. Table 1 also reveals that the kernel based classifiers generally outperform the logic based classifiers. As a result, the G^2^DE is capable of delivering satisfactory performance using a smaller number of kernel functions than the other systems.

**Table 1:** Prediction accuracies achieved by SVM, RVKDE, G^2^DE, C4.5 and RIPPER.

	Kernel based classifiers				Logic based classifiers	
		
Feature set	SVM	RVKDE	G^2^DE	G^2^DE-2	C4.5	RIPPER
1	80.17%	77.59%	80.39%	**80.60%**	77.80%	76.72%
2	**93.32%**	92.46%	92.03%	93.10%	90.95%	90.52%
3	91.60%	91.16%	91.60%	**92.46%**	91.16%	91.38%
4	78.66%	79.53%	78.66%	**80.17%**	77.37%	76.72%
Average	85.94%	85.18%	85.67%	**86.58%**	84.32%	83.84%

#kernels	361	920	6	36	10	9

The best performance among each feature set is highlighted with bold font. The G^2^DE-2 indicates the two-stage G^2^DE, which uses the first stage G^2^DE to cluster samples and than uses the second stage G^2^DE to classify each clusters. The #kernels indicate number of kernels in average, where the numbers of logic based classifiers indicate the number of rules they deliver.

In addition to compare the alternative classification algorithms, the prediction performance of the proposed method is also compared to two existing pre-miRNA identification packages, miPred [14] and miR-KDE [15]. A number (*nf*) of features from the four feature sets are selected with Wilcoxon rank sum test [37] are utilized as the feature set of G^2^DE. In current implementation, *nf *is set to seven because that the feature set yielding the best performance in Table 1 contains seven features. In this experiment, a prediction is performed by using the HU920 dataset to predict the HU464 dataset with the parameters maximizing the prediction accuracy of ten-fold cross-validation on the HU920 dataset. The five indices for binary classification (Table 2) used in miPred and miR-KDE are adopted. Table 3 shows the experimental results. G^2^DE achieves comparable performance with those delivered by miPred and miR-KDE. A notable difference between G^2^DE and miPred and miR-KDE is that G^2^DE utilizes much less kernels. G^2^DE-2 yields the best %ACC, %SE, %Fm and %MCC, which are 93.32%, 90.09%, 93.10% and 87.16%, respectively. Although the number of kernels in G^2^DE-2 is five times larger than that in G^2^DE, it is more acceptable to perform further analyses than that in miPred and miR-KDE.

**Table 2:** Evaluation measures employed in this study.

Measure	Abbreviation	Equation
Sensitivity (recall)	%SE	TP/(TP+FN)
Specificity	%SP	TN/(TN+FP)
Accuracy	%ACC	(TP+TN)/(TP+TN+FP+FN)
F-measure	%Fm	2TP/(2TP+FP+FN)
Matthews' correlation coefficient	%MCC	(TP × TN-FP × FN)/sqrt((TP+FP) × (TN+FN) × (TP+FN) × (TN+FP))

The definition of the abbreviations used: TP is the number of real pre-miRNAs detected; FN is the number of real pre-miRNAs missed; TN is the number of pseudo hairpins correctly classified; and FN is the number of pseudo hairpins incorrectly classified as pre-miRNA.

**Table 3:** Comparison of G^2^DE and two existing pre-miRNA identification packages.

Method	#kernels	%SE	%SP	%ACC	%Fm	%MCC
miPred	280	88.80%	96.55%	92.67%	92.38%	85.60%
miR-KDE	920	89.22%	96.12%	92.67%	92.41%	85.55%
G^2^DE	6	87.07%	**97.84%**	92.46%	92.03%	85.41%
G^2^DE-2	36	**90.09%**	96.55%	**93.32%**	**93.10%**	**87.16%**

The best performance among each evaluation index is highlighted with bold font. The G^2^DE-2 indicates the two-stage G^2^DE, which uses the first stage G^2^DE to cluster samples and than uses the second stage G^2^DE to classify each clusters.

### Interpretability of G^2^DE

**Figure 1:**
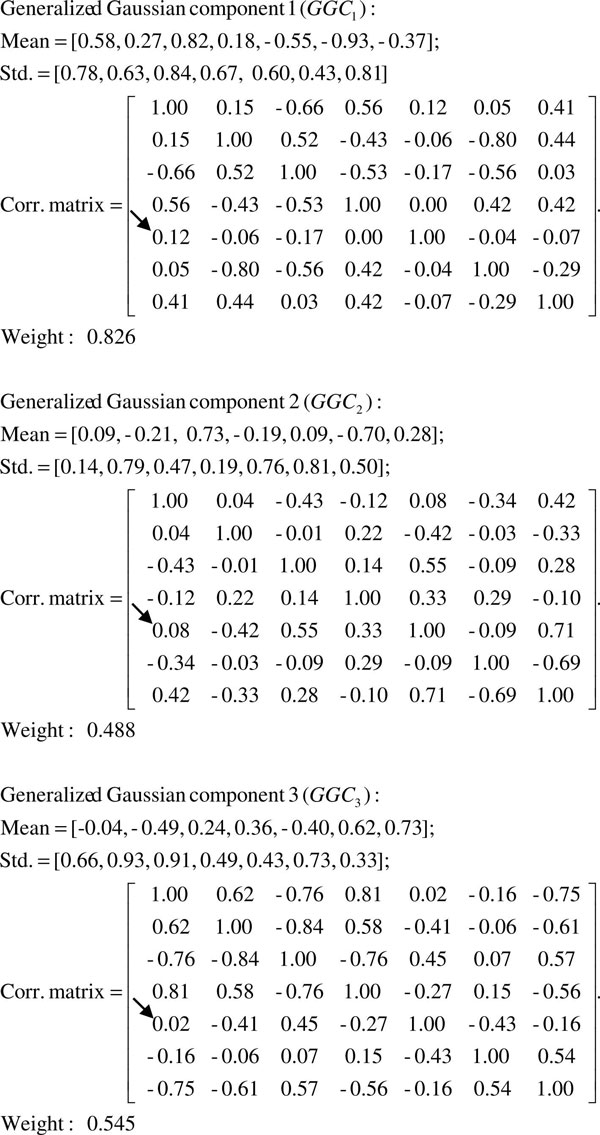
**Parameters of the three generalized Gaussian components generated by G^2^DE**. This figure shows the three generalized Gaussian components of G^2^DE with the pre-miRNAs in the HU920 dataset and the second feature set. The correlation of interest is indicated with an arrow.

**Figure 2:**
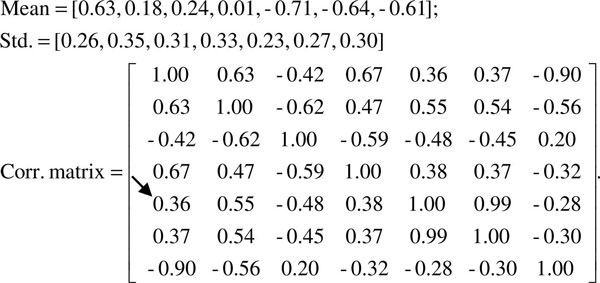
**Parameters obtained by basic statistics**. These parameters are obtained by calculating the mean, standard deviation and Pearson product-moment correlation coefficients with the pre-miRNAs of the HU920 dataset and the second feature set. The correlation of interest is indicated with an arrow.

Though the two-stage G^2^DE achieves the best performance in Table 1, the small differences to other classifiers suggests that pre-miRNA prediction algorithms have reached the maximum with current feature sets. Hence, how to interpret the learnt model of machine learning techniques for users is crucial in pre-miRNA prediction. In this subsection, the second feature set is used as an example to explain how to interpret the models generated by the G^2^DE based predictor. Figure 1 shows the parameters associated with the three Gaussian components used to summarize the pre-miRNAs in the HU920 dataset. To analyze these parameters, we compare them to the Pearson product-moment correlation coefficients (PMCC) [38] of the pre-miRNAs in the HU920 dataset (Figure 2). Parameters in the models generated by G^2^DE that differ more from the corresponding elements obtained by PMCC are more of our interest. For instance, in Figure 2, the correlation between the first feature (*mfe2*) and the fifth feature (*dQ*) of this feature set is 0.36. See the 'Feature set' subsection for detailed explanations of these features. On the other hand, the correlations between the two features in the three Gaussian components (shown in Figure 1) are 0.12, 0.08 and 0.02, all of which are relatively lower than 0.36. As PMCC summarizes the distribution of all pre-miRNAs, this analysis suggests that the HU920 dataset is composed of multiple clusters of samples, where the relation between *mfe2 *and *dQ *varies in different clusters and causes the inconsistency of correlations.

**Figure 3:**
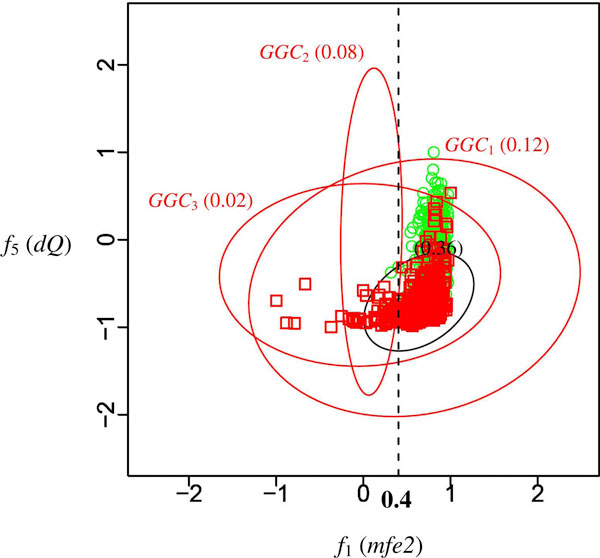
**Distribution of the HU920 dataset**. The *x*-axis is the first feature of the second feature set, ratio of MFE to the number of stems; the *y*-axis is the fifth feature of the second feature set, adjusted Shannon entropy. Red ellipses represent the generalized Gaussian components shown in Figure 1; the black ellipse represents the Gaussian component shown in Figure 2. The red squares and green circles represent the pre-miRNAs and the pseudo hairpins, respectively. Values within the parentheses indicate the correlations between these two features in the corresponding Gaussian components.

To verify the above analysis, this study depicts the HU920 dataset with their *mfe2 *and *dQ *values (Figure 3). In Figure 3, the red squares and green circles represent the pre-miRNAs and the pseudo hairpins, respectively. The red ellipses, named *GGC*_1_, *GGC*_2 _and *GGC*_3_, are the generalized Gaussian components shown in Figure 1, and the black ellipse is the Gaussian distribution shown in Figure 2. Figure 3 reveals that there are potentially two clusters of pre-miRNAs in this dataset and form a shape of a mirrored 'L' in the feature space of *mfe2 *and *dQ*. *mfe2 *measures the energy of folding while *dQ *measures the arrangement of base paring. Table 4 shows the detailed descriptions of these features. Figure 3 suggests that if a RNA sequence has low folding energy, it is probably a pre-miRNA regardless of the arrangement of its base paring. On the other hand, there is another cluster of pre-miRNAs that have similar folding energies to those of pseudo hairpins. No obvious correlation exists in both clusters of pre-miRNAs. These findings coincide with the analysis based on the models generated by G^2^DE. In this example, the Gaussian components learnt by G^2^DE help users to identify features of interest without plotting all pairs of features along with the relations between them.

**Table 4:** Summary of the adopted feature sets.

Feature	Description
Set 1	
*AA*, *AC*, ..., *UU*	Frequencies of 16 dinucleotide pairs
*%G+C*	Percentage of nitrogenous bases which are either G or C
Set 2	
*mfe2*	Ratio of *dG *to the number of stems
*mfe1*	Ratio of *dG *to *%G+C*
*dP*	Adjusted base pairing propensity. *dP *is the number of base pairs observed in the secondary structure divided by the sequence length.
*dG*	Adjusted minimum free energy of folding. *dG *is the minimum free energy (MFE) divided by the sequence length.
*dQ*	Adjusted Shannon entropy. *dQ *measures the entropy of the base pairing probability distribution (BPPD).
*dD*	Adjusted base pair distance. *dD *measures the average distance between all base pairs of structures inferred from the sequence.
*dF*	Compactness of the tree-graph representation of the sequence.
Set 3	
*zG*, *zQ*, *zD*, *zP*, *zF*	5 normalized variants of *dP*, *dG*, *dQ*, *dD *and *dF*
Set 4	
*lH*	Hairpin length
*lL*	Loop length
*lC*	Consecutive base-pairs
*%L*	Ratio of loop length to hairpin length

The table shows the order of a feature within the feature set. For example, the fifth feature in the second feature set is *dQ*.

Another useful analysis provided by the learnt model of the G^2^DE based predictor is sub-class detection. By defining that a sample *belongs *to the Gaussian component reporting the maximum function value at the location of that sample, the learnt Gaussian components of G^2^DE suggests that "a sample that belongs to *GGC*_2 _is a pre-miRNA." This statement is similar to the normal decision rule "a sample that has *mfe2 *< 0.4 is a pre-miRNA," except that the conditions (belong vs. *mfe2 *< 0.4) within the rule inferred by G^2^DE is non-linear. This non-linearity of a single rule is an important feature of G^2^DE to describe more complicated model than traditional logic based classifiers when the number of kernels of G^2^DE is limited to the number of rules of logic based algorithms. One immediate application of the sub-class detection is to group samples into clusters and then construct a classifier for each of the clusters. The performance improved by applying such two-stage framework has been shown in the previous subsection.

## Conclusion

Software predictors that identify pre-miRNAs in genomic sequences have been exploited by biologists to facilitate molecular biology research in recent years. However, design of advanced predictors of pre-miRNAs has focused mostly on coding the distinguishable sequence as well as structure characteristics of miRNAs. The study presented in this article addresses this issue from the aspect of exploiting advanced machine learning algorithms. The G^2^DE employed in this study has been designed to deliver prediction accuracy comparable with the state-of-the-art kernel based machine learning algorithms, while providing the user with good interpretability. As demonstrated by the experiments reported in this study, the models generated by the G^2^DE based classifier provide the user with crucial clues about the different characteristics of the sequences of pre-miRNAs.

## Methods

### Feature set

This work adopts 33 characteristic features which have been shown to be useful for miRNA detection in previous studies [16-19,30,39-41]. To investigate how alternative classifiers perform when using different features, these features are grouped as four different sets according to their biochemical properties. The first feature set includes 17 sequence composition variables, which comprise frequencies of 16 dinucleotide pairs and proportion of G and C in the RNA molecule. The second feature set includes seven folding measures: Minimum Free Energy (MFE) and two of its variants [17,18,39], base pairing propensity [16], Shannon entropy [18], base pair distance [18,40] and degree of compactness [19,41]. The third feature set uses the Z-score [42] to normalize the features in the second feature set except the two MFE variants. The fourth feature set includes four stem-loop features: hairpin length [15], loop length [15], consecutive base-pairs [15] and the ratio of loop length to hairpin length [15]. Table 4 shows a summary of these features.

### Dataset

The process of data preparation is the same as that in the compared pre-miRNA identification packages [14,15] for a fair comparison. 692 human miRNA precursors are collected from the miRBase registry database [43] (release 12.0) as the positive set. For the negative set, 8494 pseudo hairpins collected from the protein-coding regions (CDSs) according to RefSeq [44] and UCSC refGene [45] annotations are analyzed. These RNA sequences are extracted from genomic regions where no experimentally validated splicing event has been reported [12]. The secondary structures of the 8494 RNA sequences are obtained by executing RNAfold [46]. RNA sequences with <18 base pairs on the stem, MFE > -25 kcal/mol and multiple loops are excluded. As a result, 3988 pseudo hairpins, which are similar to genuine pre-miRNAs in terms of length, stem-loop structure, and number of bulges but not have been reported as pre-miRNAs, are used as the negative set.

Based on the positive and negative sets, one training set and one testing set are built to evaluate the pre-miRNA predictors. The training set, HU920, comprises 460 human pre-miRNAs and 460 pseudo hairpins randomly selected from the positive and negative sets, respectively. The HU920 dataset is used for parameter selection and model construction of the pre-miRNA predictors. The testing set, HU464, comprises the remaining 232 human pre-miRNAs and 232 randomly selected pseudo hairpins. Care has been taken to ensure that no pseudo hairpin is included in both datasets. Before performing any experiments on these datasets, the features are rescaled linearly by the svm-scale program [47] to the interval of [-1.0, 1.0].

### Generalized Gaussian density estimator

This work transforms samples into feature vectors and then uses them to construct a generalized Gaussian density estimator (G^2^DE) [29]. A density estimator is in fact an approximate probability density function. With the G^2^DE algorithm, one approximate probability density function of the following form is generated for each class of samples:(1)

where *d *is the dimension of the vector space and *w*_*i*_, **μ**_*i*_, and **Σ**_*i *_are the weight, mean vector, and the covariance matrix of the *i*-th Gaussian component. Let  denote the approximate probability density function for the *j*-th class of samples. Then, a query sample located at **v **is predicted to belong to the class of which the corresponding likelihood function defined in the following gives the maximum value:

where *S*_*j *_is the set of class-*j *training samples and |*S*_*j*_| is the number of class-*j *training samples.

In current G^2^DE implementation, the user can specify the maximum number of generalized Gaussian components that can be incorporated to generate the approximate probability density function for one class of samples. If the user sets this number to *k *and the total number of features of the data set is *d*, then the learning algorithm of G^2^DE needs to figure out the optimal combination of the values of the following *k*(*d*+2)(*d*+1)/2 parameters in order to generate one approximate probability density function: *k d*-dimensional vectors as the means of the generalized Gaussian components; *k *sets of *d*(*d*+1)/2 coefficients with each corresponding to the covariance matrix of one generalized Gaussian components; *k *weights. The optimal combination of parameter values are figured out using the Ranking-based Adaptive Mutation Evolutionary (RAME) algorithm [31].

In the evolutionary optimization algorithm, the objective function to be maximized is as follows:(2)

where

(1) **Z **is the vector formed by concatenating all the *k*(*d*+2)(*d*+1)/2 parameters associated with the approximate probability density function of one class of samples;

(2) #*Correct *is the number of correctly classified training samples;

(3) θ is a user-defined parameter;

(4) , where **s**_*i *_is the *i*-th training sample of class *j*.

The objective function adopted in the learning process of G^2^DE consists of two terms. Both terms have specific mathematical meanings. Maximizing term *#Correct *implies that the number of training samples of which the class can be correctly predicted with the decision model is maximized. Meanwhile, maximizing the second term implies that the mixture models give the maximum likelihood with the training samples.

### Two-stage G^2^DE

The learnt model of G^2^DE is composed of a small number of Gaussian components. In this study, a sample is defined as *belonging *to the Gaussian component reporting the maximum function value at the location of that sample. The two-stage classification framework is performed by first grouping samples belonging to the same Gaussian component into clusters and then constructing a classifier for each of the clusters. In the first stage, all training samples would be submitted to G^2^DE for constructing a mixture model of generalized Gaussian components. Suppose that the learnt model contains *n*_1 _generalized Gaussian components, essentially dividing the training dataset into *n*_1 _clusters. Each training sample would then be assigned to the Gaussian component to which it belongs. In the second stage, G^2^DE is invoked *n*_1 _times to construct *n*_1 _mixture models for each cluster. If each of the learnt models in the second stage contains *n*_2 _generalized Gaussian components, the final classifier will contain *n*_1 _× *n*_2 _generalized Gaussian components.

## Competing interests

The authors declare that they have no competing interests.

## Authors' contributions

Author C.-H. Hsieh performed all calculations and analysis and drafted the manuscript. Author D. T.-H. Chang aided in design of the methodology, interpretation of the data and manuscript preparation. Author C.-H. Hsueh and C.-Y. Wu participated in the data preparation. Author Y.-J. Oyang conceived the design of G^2^DE classifier. All authors have read and approved this manuscript.

## References

[B1] Bartel DP MicroRNAs: Genomics, biogenesis, mechanism, and function Cell 2004 116 2 281 297 10.1016/S0092-8674(04)00045-5 14744438

[B2] Lee RC Feinbaum RL Ambros V The C-Elegans Heterochronic Gene Lin-4 Encodes Small RNAs with Antisense Complementarity to Lin-14 Cell 1993 75 5 843 854 10.1016/0092-8674(93)90529-Y 8252621

[B3] Reinhart BJ Slack FJ Basson M Pasquinelli AE Bettinger JC Rougvie AE Horvitz HR Ruvkun G The 21-nucleotide let-7 RNA regulates developmental timing in Caenorhabditis elegans Nature 2000 403 6772 901 906 10.1038/35002607 10706289

[B4] Griffiths-Jones S Saini HK van Dongen S Enright AJ miRBase: tools for microRNA genomics Nucleic Acids Res 2008 36 D154 D158 10.1093/nar/gkm952 17991681 PMC2238936

[B5] Chen PY Manninga H Slanchev K Chien MC Russo JJ Ju JY Sheridan R John B Marks DS Gaidatzis D The developmental miRNA profiles of zebrafish as determined by small RNA cloning Genes & Development 2005 19 11 1288 1293 10.1101/gad.1310605 15937218 PMC1142552

[B6] Berezikov E Cuppen E Plasterk RHA Approaches to microRNA discovery Nature Genetics 2006 38 S2 S7 10.1038/ng1794 16736019

[B7] Boffelli D McAuliffe J Ovcharenko D Lewis KD Ovcharenko I Pachter L Rubin EM Phylogenetic shadowing of primate sequences to find functional regions of the human genome Science 2003 299 5611 1391 1394 10.1126/science.1081331 12610304

[B8] Grad Y Aach J Hayes GD Reinhart BJ Church GM Ruvkun G Kim J Computational and experimental identification of C-elegans microRNAs Molecular Cell 2003 11 5 1253 1263 10.1016/S1097-2765(03)00153-9 12769849

[B9] Bentwich I Avniel A Karov Y Aharonov R Gilad S Barad O Barzilai A Einat P Einav U Meiri E Identification of hundreds of conserved and nonconserved human microRNAs Nature Genetics 2005 37 7 766 770 10.1038/ng1590 15965474

[B10] Berezikov E Guryev V Belt J van de Wienholds E Plasterk RHA Cuppen E Phylogenetic shadowing and computational identification of human microRNA genes Cell 2005 120 1 21 24 10.1016/j.cell.2004.12.031 15652478

[B11] Sewer A Paul N Landgraf P Aravin A Pfeffer S Brownstein MJ Tuschl T van Nimwegen E Zavolan M Identification of clustered microRNAs using an ab initio prediction method BMC Bioinformatics 2005 6 10.1186/1471-2105-6-267 16274478 PMC1315341

[B12] Xue CH Li F He T Liu GP Li YD Zhang XG Classification of real and pseudo microRNA precursors using local structure-sequence features and support vector machine BMC Bioinformatics 2005 6 10.1186/1471-2105-6-310 16381612 PMC1360673

[B13] Brameier M Wiuf C Ab initio identification of human microRNAs based on structure motifs BMC Bioinformatics 2007 8 10.1186/1471-2105-8-478 18088431 PMC2238772

[B14] Kwang Loong S Mishra SK De novo SVM classification of precursor microRNAs from genomic pseudo hairpins using global and intrinsic folding measures Bioinformatics 2007 23 11 1321 1330 10.1093/bioinformatics/btm026 17267435

[B15] Chang DTH Wang CC Chen JW Using a kernel density estimation based classifier to predict species-specific microRNA precursors BMC Bioinformatics 2008 9 10.1186/1471-2105-9-432 PMC2638167 19091019

[B16] Schultes EA Hraber PT LaBean TH Estimating the contributions of selection and self-organization in RNA secondary structure J Mol Evol 1999 49 1 76 83 10.1007/PL00006536 10368436

[B17] Zhang BH Pan XP Cox SB Cobb GP Anderson TA Evidence that miRNAs are different from other RNAs Cell Mol Life Sci 2006 63 2 246 254 10.1007/s00018-005-5467-7 16395542 PMC11136112

[B18] Freyhult E Gardner PP Moulton V A comparison of RNA folding measures BMC Bioinformatics 2005 6 10.1186/1471-2105-6-241 PMC1274297 16202126

[B19] Gan HH Fera D Zorn J Shiffeldrim N Tang M Laserson U Kim N Schlick T RAG: RNA-As-Graphs database - concepts, analysis, and features Bioinformatics 2004 20 8 1285 1291 10.1093/bioinformatics/bth084 14962931

[B20] Nam JW Shin KR Han JJ Lee Y Kim VN Zhang BT Human microRNA prediction through a probabilistic co-learning model of sequence and structure Nucleic Acids Res 2005 33 11 3570 3581 10.1093/nar/gki668 15987789 PMC1159118

[B21] Terai G Komori T Asai K Kin T miRRim: A novel system to find conserved miRNAs with high sensitivity and specificity RNA-a Publication of the RNA Society 2007 13 12 2081 2090 10.1261/rna.655107 PMC2080609 17959929

[B22] Yang YC Wang YP Li KB MiRTif: a support vector machine-based microRNA target interaction filter BMC Bioinformatics 2008 9 10.1186/1471-2105-9-S12-S4 PMC2638144 19091027

[B23] Batuwita R Palade V microPred: effective classification of pre-miRNAs for human miRNA gene prediction Bioinformatics 2009 25 8 989 995 10.1093/bioinformatics/btp107 19233894

[B24] Jain AK Duin RPW Mao JC Statistical pattern recognition: A review IEEE Transactions on Pattern Analysis and Machine Intelligence 2000 22 1 4 37 10.1109/34.824819

[B25] Huang LT Gromiha MM Ho SY iPTREE-STAB: interpretable decision tree based method for predicting protein stability changes upon mutations Bioinformatics 2007 23 10 1292 1293 10.1093/bioinformatics/btm100 17379687

[B26] Zhou XF Ruan JH Wang GD Zhang WX Characterization and identification of microRNA core promoters in four model species PLoS Comput Biol 2007 3 3 412 423 10.1371/journal.pcbi.0030037 PMC1817659 17352530

[B27] Ho SY Hsieh CH Chen HM Huang HL Interpretable gene expression classifier with an accurate and compact fuzzy rule base for microarray data analysis Biosystems 2006 85 3 165 176 10.1016/j.biosystems.2006.01.002 16490299

[B28] Zhou GD Recognizing names in biomedical texts using mutual information independence model and SVM plus sigmoid Int J Med Inf 2006 75 6 456 467 10.1016/j.ijmedinf.2005.06.012 16112894

[B29] Hsieh C-H Chang DT-H Oyang Y-J Data Classification with a Generalized Gaussian Components based Density Estimation Algorithm International Joint Conference on Neural Networks. Atlanta, Georgia 2009

[B30] Ritchie W Legendre M Gautheret D RNA stem-loops: To be or not to be cleaved by RNAse III RNA 2007 13 4 457 462 10.1261/rna.366507 17299129 PMC1831864

[B31] Chang DTH Oyang YJ Lin JH MEDock: a web server for efficient prediction of ligand binding sites based on a novel optimization algorithm Nucleic Acids Res 2005 33 W233 W238 10.1093/nar/gki586 15991337 PMC1160262

[B32] Oyang YJ Hwang SC Ou YY Chen CY Chen ZW Data classification with radial basis function networks based on a novel kernel density estimation algorithm IEEE Transactions on Neural Networks 2005 16 1 225 236 10.1109/TNN.2004.836229 15732402

[B33] Han LY Cai CZ Lo SL Chung MCM Chen YZ Prediction of RNA-binding proteins from primary sequence by a support vector machine approach RNA 2004 10 3 355 368 10.1261/rna.5890304 14970381 PMC1370931

[B34] Dror G Sorek R Shamir R Accurate identification of alternatively spliced exons using support vector machine Bioinformatics 2005 21 7 897 901 10.1093/bioinformatics/bti132 15531599

[B35] Quinlan JR C4.5: Programs for Machine Learning 1993 San Francisco: Morgan Kaufmann

[B36] Cohen WW Fast effective rule induction International Conference on Machine Learning 1995 1995 115 123

[B37] Wilcoxon F Individual Comparisons by Ranking Methods Biometrics Bulletin 1945 1 6 80 83 10.2307/3001968

[B38] Hogg RV Tanis EA Probability and statistical inference 2006 7 Upper Saddle River, NJ: Pearson Prentice Hall

[B39] Seffens W Digby D mRNAs have greater negative folding free energies than shuffled or codon choice randomized sequences Nucleic Acids Res 1999 27 7 1578 1584 10.1093/nar/27.7.1578 10075987 PMC148359

[B40] Moulton V Zuker M Steel M Pointon R Penny D Metrics on RNA secondary structures J Comput Biol 2000 7 1-2 277 292 10.1089/10665270050081522 10890402

[B41] Fera D Kim N Shiffeldrim N Zorn J Laserson U Gan HH Schlick T RAG: RNA-As-Graphs web resource BMC Bioinformatics 2004 5 10.1186/1471-2105-5-88 15238163 PMC471545

[B42] Larsen RJ Marx ML An Introduction to Mathematical Statistics and Its Applications 2005 3 Prentice Hall

[B43] Griffiths-Jones S Grocock RJ van Dongen S Bateman A Enright AJ miRBase: microRNA sequences, targets and gene nomenclature Nucleic Acids Res 2006 34 D140 D144 10.1093/nar/gkj112 16381832 PMC1347474

[B44] Pruitt KD Maglott DR RefSeq and LocusLink: NCBI gene-centered resources Nucleic Acids Res 2001 29 1 137 140 10.1093/nar/29.1.137 11125071 PMC29787

[B45] Karolchik D Baertsch R Diekhans M Furey TS Hinrichs A Lu YT Roskin KM Schwartz M Sugnet CW Thomas DJ The UCSC Genome Browser Database Nucleic Acids Res 2003 31 1 51 54 10.1093/nar/gkg129 12519945 PMC165576

[B46] Hofacker IL Vienna RNA secondary structure server Nucleic Acids Res 2003 31 13 3429 3431 10.1093/nar/gkg599 12824340 PMC169005

[B47] Chang CC Lin CJ LIBSVM: a library for support vector machines 2001 http://www.csie.ntu.edu.tw/~cjlin/libsvm

